# Laparoscopic radical cystectomy with intracorporeal ileal conduit: one center experience and clinical outcomes

**DOI:** 10.1590/S1677-5538.IBJU.2018.0262

**Published:** 2019-07-27

**Authors:** Jianye Li, Feiya Yang, Qingbao He, Mingshuai Wang, Nianzeng Xing

**Affiliations:** 1 Department of Urology, “Beijing Chao-Yang Hospital, Capital Medical University, Beijing 100020, P.R. China

**Keywords:** Laparoscopy, Urinary Diversion, Cystectomy

## Abstract

**Purpose:**

To introduce our experience with intracorporeal ileal conduit and evaluate the safety and feasibility of this endoscopic urinary diversion.

**Materials and Methods:**

Between March 2014 and July 2017, thirty-six consecutive patients underwent laparoscopic radical cystectomy with intracorporeal ileal conduit. Patients’ demographic data, perioperative data, 90-days postoperative outcomes and complications were collected. This cohort were divided into two groups of 18 patients each by chronological order of the operations to facilitate comparison of clinical data. Data were evaluated using the students’ T test, Mann-Whitney test and Fisher's Exact test.

**Results:**

All surgeries were completed successfully with no conversion. Median total operating time and median intracorporeal urinary diversion time were 304 and 105 minutes, respectively. Median estimated blood loss was 200 mL, and median lymph node yield was 21. Twenty-six Clavien grade < 3 complications occurred within 30-days and 9 occurred within 30-90 days. Five Clavien grade 3-5 complications occurred within 30 days. No statistically significant differences were found between the two groups except for intracorporeal urinary diversion time. At median follow-up of 17.5 (range 3-42) months, 6 patients experienced tumor recurrence/metastasis and 4 of these patients died.

**Conclusions:**

Intracorporeal ileal conduit following laparoscopic radical cystectomy is safe, feasible and reproducible. With the accumulation of experience, the operation time can be controlled at a satisfactory level.

## INTRODUCTION

Radical cystectomy and urinary diversion is the gold standard treatment for muscle-invasive or high-risk superficial bladder cancer. Since laparoscopic surgery can achieve therapeutic effects equivalent to the conventional open surgery (1, 2), an increasing number of urologists choose to perform laparoscopic radical cystectomy (LRC). Urinary diversion such as ileal conduit, which is still the mainstream method, is usually performed extracorporeally through a midline incision. The use of intracorporeal urinary diversion (ICUD) is not yet widespread because of the technical difficulties of the procedure and the lengthy operating time. Thanks to the development of equipment such as the 3-D laparoscopic system and robot-assisted laparoscopy, more and more reports of ICUD have been published recently (3, 4). In the past 3 years, our surgical team has performed LRC with intracorporeal ileal conduit in 36 patients using the 3-D laparoscopic system. We also tried to simplify the procedure to make it more reproducible. In the present study, we report our experience with the surgical procedures, including procedural improvements and outcomes.

## MATERIALS AND METHODS

### Patients

Between March 2014 and July 2017, 36 consecutive patients with pathologically confirmed bladder cancer underwent LRC with the intracorporeal ileal conduit. The inclusion criteria included (1) muscle-invasive bladder cancer T2--4a, N0-Nx, M0; (2) T1G3/high-grade or high-risk and recurrent non-muscle-invasive bladder cancer; and (3) BCG-resistant Tis. The exclusion criteria mainly included distant metastases, severe heart and/or respiratory failure, severe coagulation disorders, severely insufficient renal function and history of extensive intestinal surgery. The surgical program has been approved by the Ethics Committee of Beijing Chaoyang Hospital in 2014. All patients or their agents have provided signed surgical informed consent. The clinical and perioperative data were collected prospectively. All patients were divided into 2 groups of 18 patients each in chronological order of the operations to facilitate comparison of clinical data and assessment of the development of our surgical technique, so as to clarify whether the operation time and complications rates can be controlled at a satisfactory level with the accumulation of surgical experience and the perfection of techniques. Complications occurring within ≤ 30-days and 30-90 days were recorded and categorized using the modified Clavien system (5).

### Statistical analysis

T test, Mann-Whitney test and Fisher's Exact test were performed to evaluate differences in variables between groups. SPSS 19.0 (SPSS, Chicago, IL, USA) was used for all statistical analysis, and P<0.05 was considered as statistically significant.

### Surgical Techniques

General anesthesia was administered to the patients using tracheal intubation. The patient was placed in a supine steep Trendelenburg position, and a nasogastric tube was inserted. An 18 Fr Foley catheter was positioned and 40 mg epirubicin was perfused immediately for intravesical chemotherapy before the operation.

**Figure 1:**
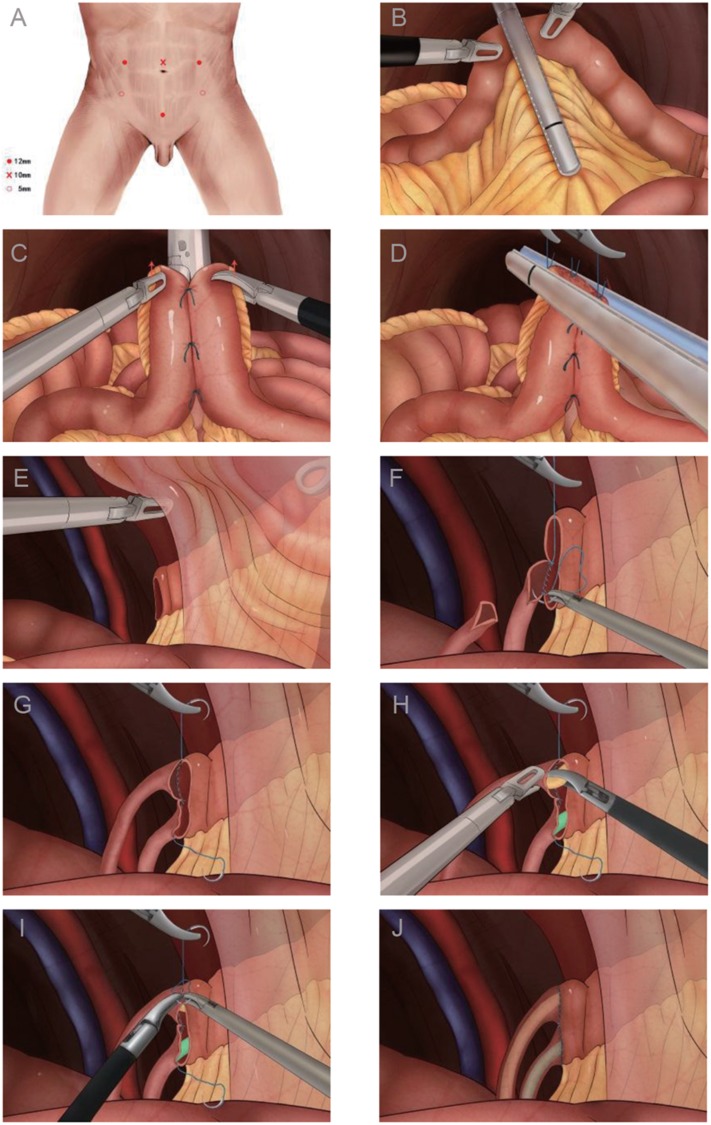
Procedures for intracorporeal ileal conduit urinary diversion. **A)** Port placement for laparoscopic radical cystectomy with intracorporeal ileal conduit. **B)** Interception of the bowel using Endo-GIA stapler. **C, D)** Restoration of ileum continuity. **E)** The distal three - quarters of the conduit were pulled into the interspace between the parietal peritoneum and the transverse abdominal muscle, by which making the conduit stable. **F, G)** The distal ureters were anastomosed with the proximal ileal conduit end-to-end independently. **H)** After half of the sutures have been placed, the 7F single J ureteric stents were inserted through the stoma and conduit into the ureters and renal pelvis. **I)** The other half of end-to-end anastomosis of the ureters-ileal conduit was completed by continuous suture. **J)** Completion of intracorporeal ureter-ileal conduit anastomosis.

Pneumoperitoneum was obtained with a Veress needle. A primary 10 mm port was placed 3 cm above the umbilicus. After inspection of the abdominal cavity, four other ports were placed in a fan-shape. Two 12 mm ports were placed on bilateral edges of the rectus abdominis, 3 cm above the umbilical level. Two 5 mm ports were placed at symmetric positions 2-3 cm superior and medial to the anterior superior iliac spines. One 12 mm port was placed at medioventral line 3cm above the pubic symphysis at the time of urinary diversion (Figure-1A).

Description of LRC procedures has been published previously (6). For male patients, the bladder and prostate were put into a specimen bag and left in the abdominal cavity temporarily. For female patients, the uterus and part of the *paries anterior vaginase* were excised and put into the specimen bag with the bladder, which was extracted vaginally. The vaginal stump was sutured. After LRC had been accomplished, extended pelvic lymph node dissection (ePLND) was performed. The lymph nodes (LNs) in the level of aortic bifurcation and inferior mesenteric artery were dissected if computed tomography (CT) or magnetic resonance imaging (MRI) indicated or if intumescent LNs in bifurcation common iliac vessels level were observed during the surgery.

After performing appendectomy, a 12 mm trocar was placed at medioventral line 3 cm above the pubic symphysis. A 15-20 cm ileum segment was identified approximately 25 cm away from the ileocecal junction as the conduit. A 60 mm Endo-GIA stapler was used to divide the bowel lumen on both sides of the conduit (Figure-1B). When restoring the ileum continuity, an interrupted suture for 3 needles (3-0 polyglactin 910) was made on the respective serous membrane of anti-mesenteric borders of the two ileum loops. Side-to-side ileal continuity was restored by one firing of the 60 mm Endo-GIA stapler along the respective anti-mesenteric borders (Figure-1C). The open end of the joined ileum was closed by the transverse firing of an Endo-GIA stapler (Figure-1D). The mesenteric window was closed to prevent internal hernia. A channel was made by separating the tissues between the sacroiliac and the sigmoid mesentery, and the left ureter was mobilized cephalad and delivered to the right side through this channel. Before performing ureter-ileal conduit anastomosis, the peritoneum viscerale of the right lower abdominal wall was incised. The skin and abdominal wall muscle were incised on the stoma site marked previously. The parietal peritoneum of the right lower abdomen is separated from the transverse abdominal muscle, so an interspace is created between them. The distal three-quarters of the conduit were pulled into this interspace by an oval clamp through the pre-designed stoma site to stabilize the conduit (Figure-1E). The distal ends of the ureters were incised and spatulated by laparoscopic scissors for a distance of approximately 1.5 cm. The proximal end of the conduit was incised and then the end-to-end ureter-ileal conduit anastomosis was performed (4-0 polyglactin 910) (Figures 1F and G). After half of the sutures have been placed, the 7F single J ureteric stents were inserted through the stoma and conduit into the ureters and renal pelvis (Figure-1H), then the other half of end-to-end anastomosis of the ureters-ileal conduit was completed by continuous suture (Figures 1I and J). The creation of a stoma at skin level was finished using conventional techniques. For male patients, the specimen bag was extracted through a small abdominal incision by extending the 12 mm port 3 cm above the pubic symphysis.

## RESULTS

Patients’ demographics are shown in Table-1. A total of 36 patients underwent the procedure, including 26 men and 10 women. Seven patients received preoperative neoadjuvant chemotherapy. Nineteen patients received transurethral resection of bladder tumor (TURBT). Preoperative pathological examination showed 1 patient with squamous cell carcinoma and 2 with adenocarcinoma. All surgeries were performed success fully, and no patients were converted to extracorporeal urinary diversion (ECUD).

**Table 1:** Patient characteristics.

Total no. of patients	36
Male (%)	26 (72.2)
Female (%)	10 (27.8)
Age, y, mean ± SD (range)	63.7±9.6 (42-83)
BMI, kg/m^2^, mean ± SD (range)	24.7±3.0 (18.8-31.1)
Smoking history (%)	14 (38.9)
**ASA score (%)**	
1	8 (22.2)
2	25 (69.4)
3	3 (8.3)
4	0
**ECOG score (%)**	
≤ 2	36 (100)
> 2	0
Neoadjuvant chemotherapy (%)	7 (19.4)
Previous TURBT (%)	19 (52.8)
**Preoperative T stage (%)**	
Tis	1 (2.8)
T1	9 (25)
T2	9 (25)
T3	13 (36.1)
T4	4 (11.1)
**Preoperative grade (%)**	
Low	4 (11.1)
High	29 (80.6)
Squamous cell carcinoma	1 (2.8)
Adenocarcinoma	2 (5.6)

**BMI** = body mass index; **ASA** = American Society of Anesthesiologists (classification); **ECOG** = Eastern Cooperative Oncology Group; **TURBT** = Transurethral resection of bladder tumor.

Table-2 shows patients’ perioperative characteristics and pathology. Two patients had satisfied preoperative sexual function and required to protect the neurovascular bundle (NVB). Three patients had positive surgical margins. Four patients were found to have incidental prostate adenocarcinoma and 6 were found to have concomitant carcinoma *in situ* (CIS). One woman had received radical hysterectomy and ePLND due to cervical cancer more than 1 year prior. Therefore, LNs dissection was not performed for this patient and the lymph node yield (LNY) was recorded as zero. One patient was readmitted within 30 days because of pyelonephritis. A total of 40 complications occurred in 63.9% of patients (Table-3) within 90 days, of which 87.5% were minor complications (Clavien 1-2). The high-frequency complications were infection (n=11) and gastrointestinal (n=6). High-grade cardiovascular and pulmonary complications (Clavien 3-5) occurred in 5 patients, resulting in 2 deaths within 30 days. No serious ileal conduit-related complications occurred such as anastomotic stenosis, leakage or necrosis.

**Table 2:** Perioperative characteristics and pathology.

Operating time, min, median (range)		
	Total time, min	304 (180-540)
	Diversion time, min	105 (60-170)
LOS, day, median (range)		11.5 (6-25)
EBL, mL, median (range)		200 (50-1100)
IT, no. (%)		6 (16.7)
Conversions (%)		0
**NVB sparing procedures: male cystectomies (%)**		
	Non	24 (92.3)
	Unilateral	0
	Bilateral	2 (7.7)
**Postoperative pT stage (%)**		
	Tis	1 (2.8)
	T1	7 (19.4)
	T2	7 (19.4)
	T3	12 (33.3)
	T4	9 (25)
**Postoperative grade**		
	Low	2 (5.6)
	High	31 (86.1)
Squamous cell carcinoma		1 (2.8)
Adenocarcinoma		2 (5.6)
Concomitant CIS (%)		6 (16.7)
Incidental prostate		4 (11.1)
adenocarcinoma (%)		
PSM (%)		3 (8.3)
LNY, no., median (range)		21 (0-41)
**pN stage (%)**		
	N0	21 (58.3)
	N1	1 (2.8)
	N2	8 (22.2)
	N3	6 (16.7)
Time to intake of liquid diet, d, median (range)		4 (2-8)
Time to ambulation, day, median (range)		2 (1-5)

**Min** = minute; **LOS** = length of stay; **EBL** = estimated blood loss; **IT** = intraoperative transfusion; **NVB** = neurovascular bundle; **LNY** = lymph node yield; **CIS** = carcinoma *in situ*; **PSM** = positive surgical margin.

**Table 3:** The 0-30 and 31-90 days complications of patients.

Short-term complications (0-30 days)				
Complication	Definition	No. of events (%)	Treatment	Clavien grade
Paralytic ileus	No bowel sounds for 4 days after operation	6 (15)	Conservative	1
Lymphocele	CT-detected accumulation of lymph	1 (2.5)	Conservative	1
Renal insufficiency	Transient elevation of serum creatinine	1 (2.5)	Conservative	1
Anemia	Hemoglobin < 9 g/dL	4 (10)	Conservative	1
Anemia	Hemoglobin < 9 g/dL	2 (5)	Transfusion	2
Hypokalemia	Serum potassium concentration <3.5mmol/L	4 (10)	Potassium supplementation	2
FUO	Fever > 38.8 °C with unknown etiology after 7 days of surgery	3 (7.5)	Antibiotics	2
UTI	Infection of urine with positive urine culture	2 (5)	Antibiotics	2
Pyelonephritis	Infection of the upper urinary tract	(2.5)	Antibiotics	2
Hypoproteinemia	Serum albumin concentration < 25 g/L	2 (5)	Intravenous infusion of albumin	2
HF	Heart failure leading to low-output syndrome	2 (5)	Drugs	4a
ACS	Acute coronary syndrome leading to precordial region syndrome	1 (2.5)	Drugs	4a
Death		2 (5)		5
Long-term complications (31-90 days)				
Complication	Definition	No. of events (%)	Treatment	Clavien grade
Hydronephrosis	Dilatation of the upper urinary tract	3 (7.5)	Conservative	1
UTI	Infection of urine with positive urine culture	4 (10)	Antibiotics	2
Pyelonephritis	Infection of the upper urinary tract	1 (2.5)	Antibiotics	2
DVT	Deep venous thrombosis (lower limbs)	1 (2.5)	Anticoagulant	2

**CT** = computed tomography; **FUO** = fever of unknown origin; **HF** = heart failure; **ACS** = acute coronary syndrome; **RBCs** = red blood cells; **UTI** = urinary tract infection; **DVT** = deep venous thrombosis

**Figure 2:**
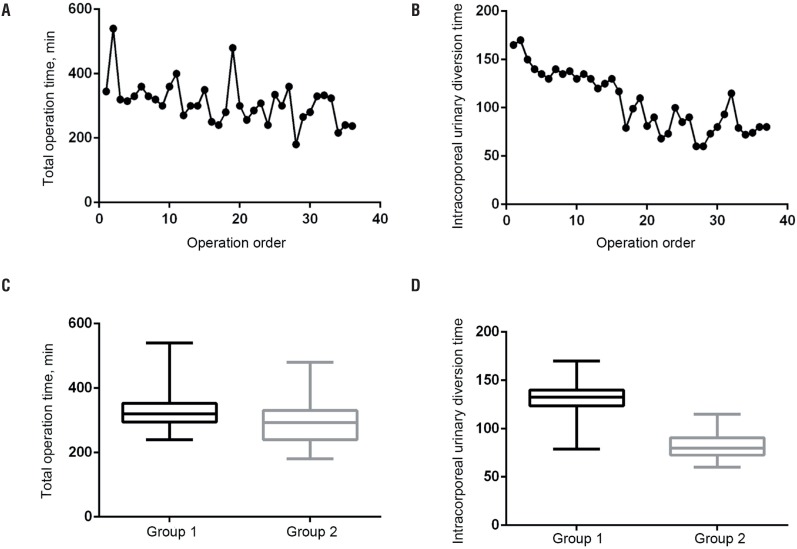
Total operation time and intracorporeal urinary diversion (ICUD) time. **A, B)** Both the total operation time and ICUD time showed a downward trend. **C)** There was no statistically significant difference in total operation time between the 2 groups. **D)** The ICUD time had the statistical difference between the 2 groups.

Demographics and operation-related data between the 2 groups are shown in Table-4. No statistically significant differences were found in total operating time, EBL, length of stay (LOS), LNY and 90-day complication rate between the two groups. Although both total operating time and ICUD time had a downward trend, only the latter had a statistically significant difference between the two groups (Figure-2), which decreased from a median of 132.5 minutes in group 1 to 80 minutes in group 2.

**Table 4:** Comparison between the two groups.

		Patients 1-18	Patients 19-36	All patients	p value
Age, years, mean ± SD (range)		64.5±9.7	62.9 ± 9.7	63.7±9.6	0.62
BMI, kg/m^2^, mean ± SD (range)		23.8±2.6	25.6 ± 3.2	24.7 ± 3.0	0.083
Total operation time, min, median		320 (240, 540)	292.5 (180, 480)	304 (180-540)	0.085
ICUD time, min, median		132.5 (79, 170)	80 (60, 115)	105 (60-170))	0.000
EBL, mL, median		175 (50, 1100)	200 (50, 650)	200 (50-1100)	0.389
**Postoperative pT stage**					**1.000**
	pT stage <T2, no. (%)	4	4	8	
	pT stage ≥T2, no. (%)	14	14	28	
LNY, no., median		17 (5,41)	22 (0,38)	21 (0,41)	0.462
LOS, d, median		10 (6, 22)	13.5 (6, 25)	11.5 (6, 25)	0.203
No. of patients					
	<30 d complications*				0.500
Clavien 0, no		6	9	15	
Clavien 1-2, no.		8	8	16	
Clavien 3-5, no.		4	1	5	
	30-90 d complications*				0.398
Clavien 0, no		12	15	27	
Clavien 1-2, no.		5	2	7	
Clavien 3-5, no.		0	0	0	

**BMI** = body mass index; **ICUD** = intracorporeal urinary diversion; **EBL** = estimated blood loss; **LOS** = length of stay; **LNY** = lymph node yield.

* Number of patients with the complications classified by Clavien system

At a median follow-up of 17.5 (range 3-42) months, 6 patients experienced tumor recurrence/metastasis and 4 of these patients died.

## DISCUSSION

Sanchez et al. (7) first reported LRC with the extracorporeal ileal conduit in 1993, and since then, LRC with ECUD was accepted by an increasing number of urologists. Currently, intracorporeal radical cystectomy and lymphadenectomy are technically established. The urinary diversion procedure is complicated and usually performed extracorporeally. To make the operation less invasive, urologists began to explore the feasibility of ICUD. In 2000 and 2002, Gill et al. (8, 9) reported successful cases of LRC with intracorporeal ileal conduit and continent orthotopic ileal neobladder. Harvest of the ileum for reconstruction of the reservoir and anastomosis of the ureter-reservoir were accomplished under laparoscopy. Subsequently, successful cases of robot-assisted radical cystectomy (RARC) with ICUD were also reported (3, 4). However, as the number of cases increased, the complexity of ICUD and longer operation time were observed to produce more complications (10, 11). Consequently, whether to continue performing ICUD was in dispute.

**Figure 3:**
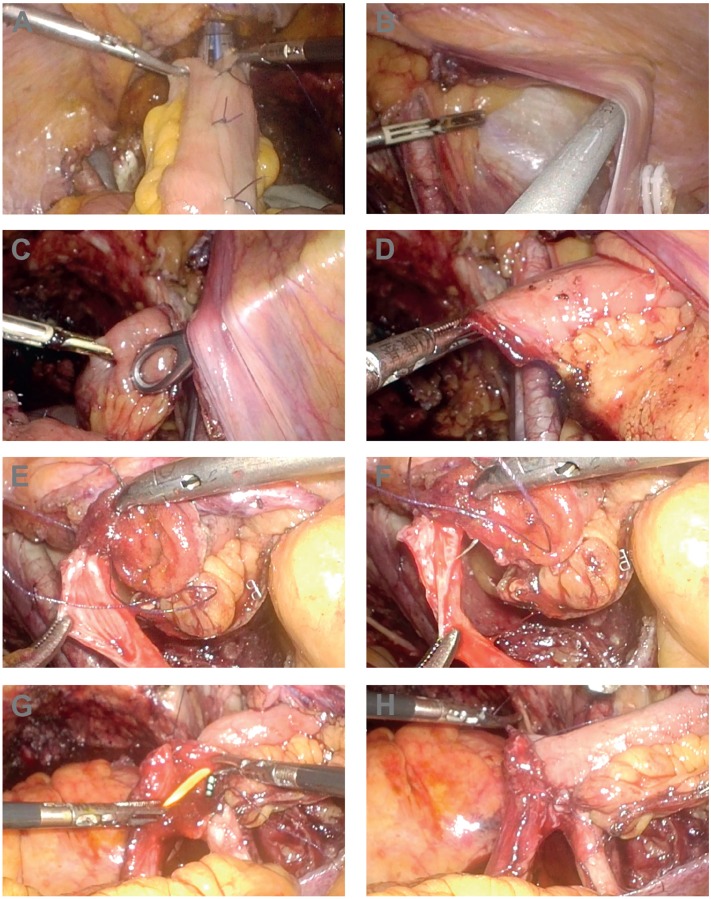
Intra-operative pictures of intracorporeal ileal conduit urinary diversion. **A)** Using the laparoscopic 60-mm Endo GIA stapler to restore intestinal continuity, the bilateral bowel ends were pulled up toward the angle of the stapler as far as possible, only one firing of stapler is enough. **B)** The parietal peritoneum of the right lower abdominal wall was dissociated from the transverse abdominal muscle, so an interspace between them was created. **C, D)** The ileal conduit was pulled through the stoma site and kept in the interspace between the peritoneum and the transverse abdominal muscle before ureter-ileal conduit anastomosis. **E-H)** The ureters and proximal enteric cavity of the conduit were anastomosed end-to-end independently.

The rapid development of laparoscopic technology, including operative and stapling instruments, made ICUD more feasible. Many surgeons proposed that appropriate surgical strategy, a reasonable choice of equipment, improved surgical techniques, and standardized surgical procedures were able to simplify ICUD, thus shortening the operating time and reducing complications (12, 13). Our surgical team began to perform LRC with the intracorporeal ileal conduit in March 2014 and modified some surgical procedures to simplify the surgery and shorten the operating time. First, when using the laparoscopic 60 mm Endo GIA stapler to restore intestinal continuity, the bilateral bowel ends were pulled up toward the angle of the stapler as far as possible (Figure-3A), then only one firing of stapler is enough to get the appropriate width of the intestinal anastomosis, not as the other literatures reported that a second fire was needed (14, 15). This improvement reduces the cost of Endo GIA stapler and shortens the operation time. Moreover, our results did not show that any patient experienced mechanical intestinal obstruction after the surgery. Secondly, fixing the ileal conduit on the abdominal wall to facilitate the ureter-ileal conduit anastomosis. Before performing ureter-ileal conduit anastomosis, we dissociated the parietal peritoneum from the transverse abdominal muscle of the right lower abdominal wall (Figure-3B). The ileal conduit was pulled through the stoma site and kept in the interspace between the peritoneum and the transverse abdominal muscle before ureter-ileal conduit anastomosis (Figures 3C and D). This procedure can stabilize the conduit and facilitates single J tube implantation and ureter-ileal anastomosis. There was also other way reported to facilitate the procedures of ICUD. Guru et al. (16) designed the “Marionette” technique: the distal end of the ileal conduit was suspended by a 152 cm 1-silk suture, which allowed for raising and lowering the conduit like a “marionette”, helping anastomose the ureters and conduit. Thirdly, we designed a new method to perform the ureter-ileal conduit anastomosis, which is the most important technical improvement of this surgery. Intracorporeal ureter-ileal conduit anastomosis is the most difficult part of the procedure; several different techniques are applied, including the Bricker (17) and Wallace (18) methods. However, these techniques are difficult to perform under laparoscopy since they were designed for open surgery. In order to simplify this procedure, we improved the anastomosis: we incise and spatulate the distal ends of the ureters and anastomose the ureters and proximal enteric cavity of the conduit end-to-end independently (Figures 3E-H). This method is simple and can decrease the operating time; most important, it is suitable for laparoscopic surgery. Both the ureter-conduit anastomosis and single J ureteric stents insertion are quite convenient under laparoscopy. This method also has a low incidence of ureter-conduit anastomosis related complications. Azzouni et al. (19) reported 100 cases of robot-assisted intracorporeal ileal conduit using ureter-conduit end-to-side anastomosis, and hydronephrosis developed in 6 patients with 9 renal units, of which 4 units needed percutaneous nephrostomy. In the present study, mild hydronephrosis was found in 3 patients at 31-90 days after the surgery, and 1 patient's hydronephrosis disappeared without treatment at 9-months follow-up. No patients needed percutaneous nephrostomy. Our results showed that the new technique led to lower incidence of anastomotic stenosis, although long-term observation is still needed. Another advantage of our technique is that the staples on the conduit were abscised, which may reduce the chance of conduit stones’ formation, as previously described (20).

Many urologists have advocated giving up ICUD because of the long operating time of ICUD, which increases the incidence of serious postoperative complications (10). Laparoscopic cystectomy and LN dissection techniques are technically advanced, so it is very hard to shorten the time of these procedures. Therefore, many urologists have focused on the technological improvements in urinary diversion designed to improve surgical efficiency (13, 19). Our technical improvements focus primarily on the main steps of ileal conduit creation. With the increasing number of operations, the surgeons’ experience accumulates gradually, and both total operating time and ICUD time showed a downward trend in operative time. In the present study, all cases were divided into 2 groups with 18 patients in each according to the order of operations, in order to facilitate comparison of clinical data and assessment of the development of surgical technique. All ICUD procedures were completed by one experienced surgeon. With the accumulation of experience and the perfection of techniques, the operative time of these procedures decreased significantly from a median of 132.5 minutes in group 1 to 80 minutes in group 2, which means that the ICUD operative time can be controlled at a satisfactory level. There was no statistical difference in total operation time between the 2 groups, the possible reason for which may be that the procedures of laparoscopic cystectomy, LN dissection, the conduit end exteriorization and stoma maturation were performed by different young surgeons in the team under the guidance of experienced surgeons, which interfered with the statistical results. The significantly shorter operative time in the Group 2 than in Group 1 indicates that the ICUD is feasible and should not be given up in terms of operative time alone.

Some medical centers have reported retrospective controlled studies comparing ICUD with previous ECUD. Recent retrospective studies have shown that ICUD had fewer complications than ECUD (21–23), although the operating time of ICUD group was not dominant, the intraoperative blood loss, blood transfusion rate and total complications rates were better than those in the ECUD group. The morbidity of gastrointestinal complications in ICUD was significantly lower than those in ECUD, which also confirmed previous assumptions that ICUD can reduce intestinal fluid loss and intestinal trauma and relieve intestinal wall edema, which can decrease the incidence of intestinal complications. The incidence of perioperative and short-term complications in our patients was acceptable. Overall, 63.9% of patients experienced complications within 90 days, of which Clavien 1-2 were the most common (87.5% of all complications). Among all complications, 27.5% were infection. Clavien 3-5 occurred in 5 patients within 30 days. No statistically significant differences were found in EBL, LOS and complication rates between the two groups.

This study also has some limitations. We report our single institution experience with LRC and ICUD but there is no control group with ECUD in the same period. The randomized clinical trial research between ICUD and ECUD is in progress and more clinical data should be collected to evaluate the long-term effects.

## CONCLUSIONS

In conclusion, regardless of the complexity of the LRC with intracorporeal ileal conduit, the procedure is safe and feasible. Improvements in surgical techniques and accumulation of surgical experience can help to shorten the operating time and make the technique reproducible.

## ABBREVIATIONS

LRC: Laparoscopic Radical Cystectomy
ECUD: Extracorporeal Urinary Diversion
ICUD: Intracorporeal Urinary Diversion
CIS: Carcinoma *in Situ*
TURBT: Transurethral Resection of Bladder Tumor
BMI: Body Mass Index
LOS: Length of Stay
EBL: Estimated Blood Loss
IT: Intraoperative Transfusion
LNY: Lymph Node Yield
LN: Lymph Node
LNs: Lymph Nodes
NVB: Neurovascular Bundle
CT: Computed Tomography
MRI: Magnetic Resonance Imaging
ASA: American Society of Anesthesiologists
ECOG: Eastern Cooperative Oncology Group
PSM: Positive Surgical Margin
FUO: Fever of Unknown Origin
HF: Heart Failure
ACS: Acute Coronary Syndrome
RBCs: Red Blood Cells
UTI: Urinary Tract Infection
DVT: Deep Venous Thrombosis
RARC: Robot-Assisted Radical Cystectomy
